# Properties of MHC Class I Presented Peptides That Enhance Immunogenicity

**DOI:** 10.1371/journal.pcbi.1003266

**Published:** 2013-10-24

**Authors:** Jorg J. A. Calis, Matt Maybeno, Jason A. Greenbaum, Daniela Weiskopf, Aruna D. De Silva, Alessandro Sette, Can Keşmir, Bjoern Peters

**Affiliations:** 1 Theoretical Biology & Bioinformatics, Utrecht University, Utrecht, The Netherlands; 2 Division of Vaccine Discovery, La Jolla Institute for Allergy and Immunology, La Jolla, California, United States of America; 3 Genetech Research Institute, Colombo, Sri Lanka; Imperial College London, United Kingdom

## Abstract

T-cells have to recognize peptides presented on MHC molecules to be activated and elicit their effector functions. Several studies demonstrate that some peptides are more immunogenic than others and therefore more likely to be T-cell epitopes. We set out to determine which properties cause such differences in immunogenicity. To this end, we collected and analyzed a large set of data describing the immunogenicity of peptides presented on various MHC-I molecules. Two main conclusions could be drawn from this analysis: First, in line with previous observations, we showed that positions P4–6 of a presented peptide are more important for immunogenicity. Second, some amino acids, especially those with large and aromatic side chains, are associated with immunogenicity. This information was combined into a simple model that was used to demonstrate that immunogenicity is, to a certain extent, predictable. This model (made available at http://tools.iedb.org/immunogenicity/) was validated with data from two independent epitope discovery studies. Interestingly, with this model we could show that T-cells are equipped to better recognize viral than human (self) peptides. After the past successful elucidation of different steps in the MHC-I presentation pathway, the identification of variables that influence immunogenicity will be an important next step in the investigation of T-cell epitopes and our understanding of cellular immune responses.

## Author Summary

T-cells have to recognize peptides presented on MHC molecules to be activated and elicit their effector functions. Some peptide-MHC-I complexes (pMHCs) are better recognized by T-cells; we call such pMHCs more immunogenic. For other pMHCs, no recognizing T-cells seem to exist; we call such pMHCs non-immunogenic. We set out to determine which properties of pMHCs cause such differences in immunogenicity, by carefully collecting a large set of immunogenic and non-immunogenic pMHCs, and analysing the difference between these sets. Two important observations were made: First, in line with previous observations, we showed that positions P4–6 of a presented peptide are more important for immunogenicity. Second, some amino acids, especially those with large and aromatic side chains, seem to be better recognized by T-cells as they associate with immunogenicity. Next, this information was combined into a simple model to predict the immunogenicity of new pMHCs (this model is made available at http://tools.iedb.org/immunogenicity/). Interestingly, with this model we could show that T-cells are equipped to strongly recognize viral peptides. After the past successful elucidation of different steps in the MHC-I presentation pathway, the identification of variables that influence immunogenicity will be an important next step in the investigation of T-cell epitopes and our understanding of cellular immune responses.

## Introduction

Peptides presented on MHC class I (MHC-I) molecules at the cell-surface are screened by CD8^+^ T-cells to detect aberrancies, such as an infection. The strength of the interaction between the peptide-MHC complexes (pMHC) and T-cell receptors (TCRs), depends both on the MHC-I molecule and the presented peptide. A specific pMHC will be recognized by an estimated average of one in 100,000 naive T-cells [1]–[4], but this precursor frequency differs for different pMHCs [3], [5], [6]. In the context of an infection, recognized pMHCs can stimulate T-cells to proliferate into an effector T-cell population that finds and kills infected cells presenting this pMHC. Such a pMHC, that is the target of a specific T-cell immune response, is called an epitope.

In past years, many efforts have been put in determining which peptides are presented on MHC-I molecules. For numerous peptide-MHC combinations the binding affinity has been measured [7], [8], and this data enabled the development of highly accurate MHC-I binding predictors [7], [9]–[15]. Furthermore, the processing of precursor proteins into MHC-I ligands by the proteasome, other proteases and the TAP transporter has been studied extensively [16]–[22], and data from these studies were used to construct successful processing-predictors [23]–[25]. Thanks to this progress, for a pathogen such as HIV-1 it is now possible to predict reliably which peptides will be presented on a certain MHC-I molecule, and test subsequently if these predicted pMHCs are epitopes [26].

Despite high accuracy predictions of which pMHCs are formed upon infection, what distinguishes epitopes from non-epitopes is still an open question. Several factors have been described that could explain the difference between epitopes and non-epitopes. First, the abundance of a pMHC plays a role in immune targeting [27]–[29], the abundance can be affected by (1) peptide-MHC binding affinity [30], pMHC (2) stability [31], (3) the abundance of the precursor protein [28], [29], [32], and (4) the efficiency of MHC ligand processing [28], [29], [33], [34]. Second, a pMHC should be recognized by T-cells, i.e. it should be immunogenic. Third, the pMHCs derived from certain proteins that are expressed early in infection are more likely to evoke a response [35], [36]. Fourth, even if an immunogenic peptide is presented under the right conditions, a response might be blocked by regulatory processes if a (nonself) pMHC is too similar to a self pMHC [37]–[39]. We recently estimated that about one-third of the nonself pMHCs is too similar to self [40]. Finally, an immune response might be outcompeted by other T-cell responses due to limited survival factors, a phenomenon called competitive exclusion [41], [42]. Thus, a plethora of effects eventually determines which peptides are epitopes.

The identification of epitopes is key to the study and understanding of cellular immune responses, and is of great importance in vaccine development. Therefore, we studied an important step that influences whether a pMHC can be an epitope: immunogenicity. In this paper, we will refer to T-cell recognized and unrecognized pMHCs as immunogenic and non-immunogenic pMHCs. Immunogenicity can be measured directly in peptide-immunization experiments, as other factors like the right processing of a peptide or the expression of a source protein are excluded from negatively influencing the T-cell response. Peptide-immunization experiments have shown that about half of the pMHCs are immunogenic [43], [44]. We collected a set of immunogenic and non-immunogenic pMHCs, and compared the amino acid frequencies in both sets. This analysis showed that T-cells have a preference for certain amino acids, especially aromatic and large residues. Next, we analyzed the importance of different positions of the presented peptides with respect to immunogenicity. As expected, the middle part of the presented peptide (P4–P6) was shown to be most important. These results were validated by combining them into a simple enrichment model and testing if this model could estimate the immunogenicity of new pMHCs. Both in cross-validations, and in two independent data sets could we validate our observations, by showing that immunogenicity is to some extent predictable (AUC = 0.65). In addition, we used the prediction model to examine a possible adaptation of the immune system to recognize pathogen-derived peptides, and showed that a preference for these peptides exists.

## Results

### Classifying immunogenic pMHCs

To investigate the peptide preferences in T-cell recognition, one needs well defined sets of immunogenic and non-immunogenic pMHCs. Therefore, strict parameters were set to classify only those pMHCs for which immunogenicity or the absence thereof was strongly shown upon infection or vaccination. The classification of immunogenic pMHCs from positive immune responses upon infection or vaccination is relatively straight-forward. In contrast, the classification of non-immunogenic (i.e. unrecognized by T-cells) pMHCs upon natural infections is difficult, as many other factors could cause the lack of an immune response besides non-immunogenicity (see Introduction). Therefore, for the classification of non-immunogenic pMHC, we required a peptide-immunization study in combination with a high predicted peptide-MHC-I binding affinity, to ensure that MHC-I presentation of the assayed peptide to T-cells was feasible. However, this strict definition excluded humans as a host for the identification of non-immunogenic pMHCs, since peptide-immunization studies have rarely been conducted in humans. Even though immunogenic pMHCs could be derived from humans, we decided to initially collect only data from mice, to avoid any bias caused by disparate sampling from different hosts. In addition, we compared only peptides presented on MHC-I molecules from the same species (H-2 or HLA, where data originate from HLA-transgenic mice), of the same length (9mers), and a redundancy reduction method was applied to avoid oversampling effects (see Methods for a detailed description on the data collection and classification process).

**Figure 1:**
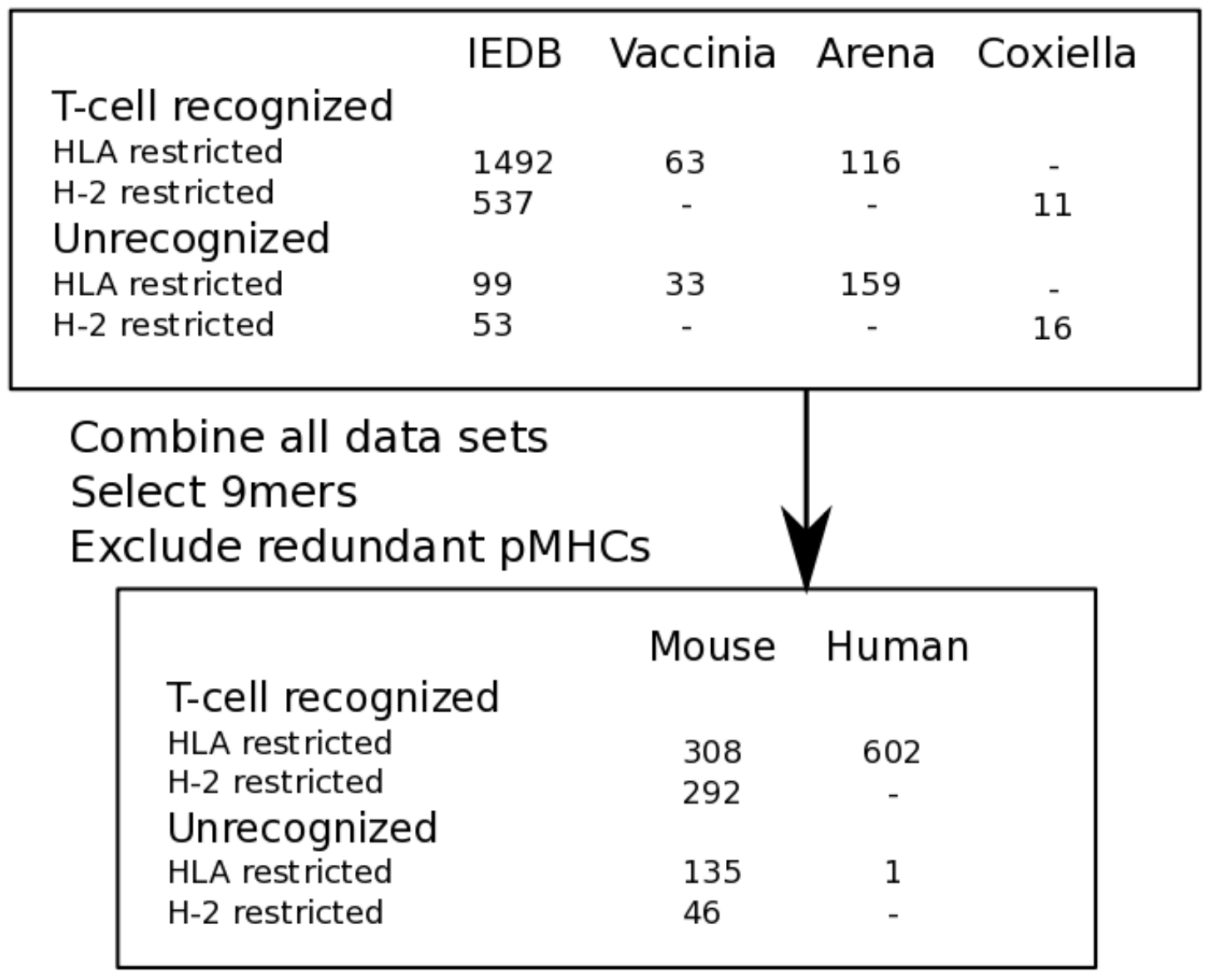
Data acquisition and handling oversight. Data was collected from four different sources (see Methods). The first panel shows how many pMHCs were derived from each data set and their respective MHC restrictions and immunogenicity status. Data from all sets was combined, the number of non-redundant 9mers with respect to the host in which the data was obtained is shown in the second panel.

Four sources of data were used, the Immune Epitope Database (IEDB) [45], and three immunogenicity studies in mice (see methods and [43], [44]). 600 Immunogenic and 181 non-immunogenic non-redundant 9mer pMHCs that fulfilled our strict criteria, were selected for further characterization (see Figure 1). This relatively large set of immunogenic and non-immunogenic pMHCs were further analyzed to determine what properties can explain the difference in immunogenicity.

### Amino acid properties of immunogenic pMHCs

**Figure 2:**
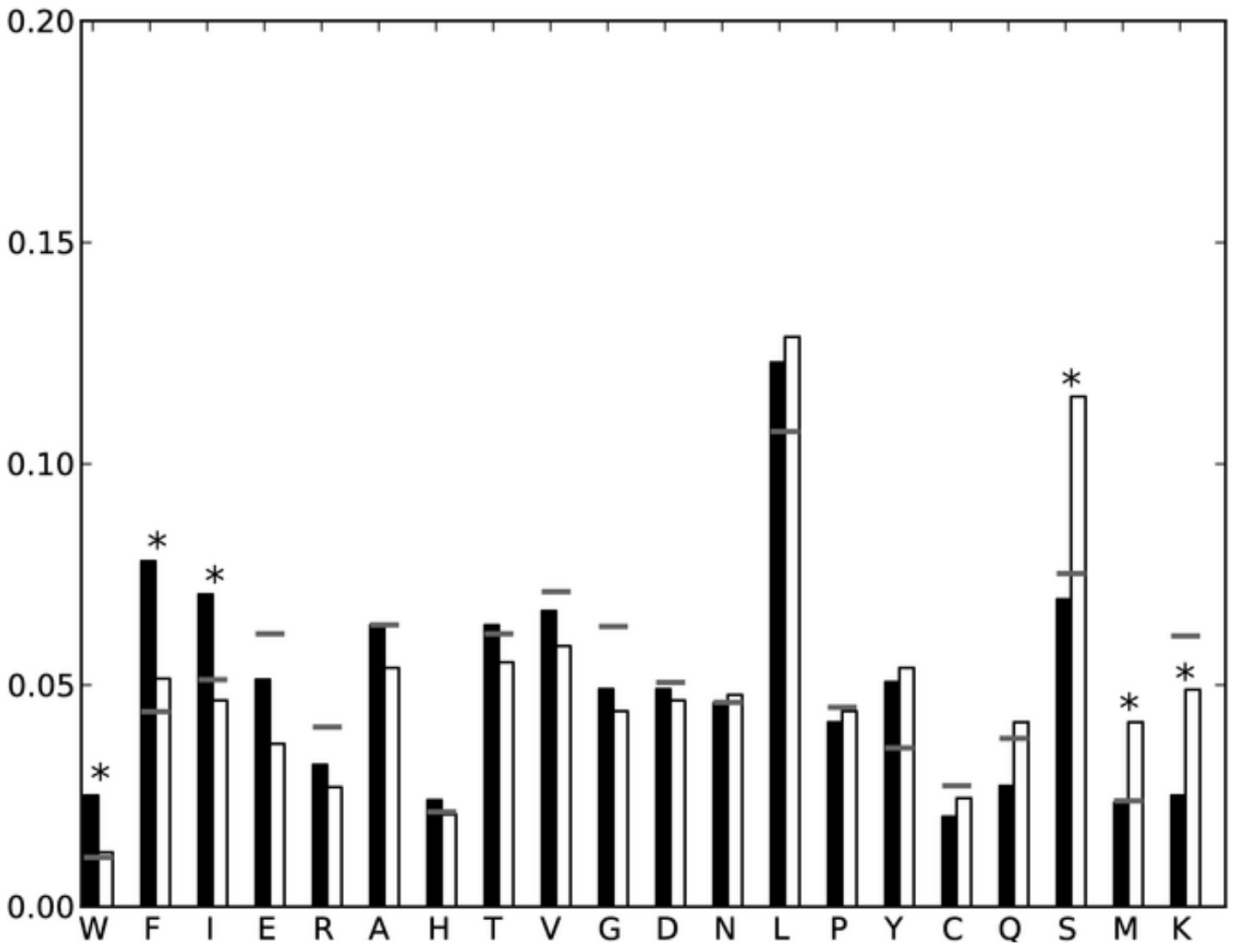
T-cell preferences for different amino acids in HLA class I presented peptides. The fraction of an amino acid in immunogenic (left bar, filled) and non-immunogenic (right bar, unfilled) peptides presented on HLA class I molecules is shown. Significantly different distributions are indicated with a star (Permutation test, see Methods: p<0.05; False discovery rate (FDR) for multiple testing determined as in [46]: q<0.05). The background frequency for each amino acid in the protein sequences that were a source of the immunogenic or non-immunogenic peptides is shown by a grey line.

The immunogenic and non-immunogenic pMHCs, classified above, can be compared to see what properties associate with immunogenicity. We hypothesize that certain amino acids are more likely to interact with TCRs, and therefore increase the immunogenicity of a pMHC. Conversely, some amino acids could abolish TCR interactions. To test this hypothesis, per amino acid the association with immunogenicity was tested, and a comparison with background amino acid frequencies was made. To prevent any bias that might rise due to the binding motif of MHC-I molecules, residues at positions with an influence on the binding affinity were excluded from the analysis (see Methods). In addition, all peptides in our data set, i.e. immunogenic and non-immunogenic ones, were required to have a predicted binding affinity stronger than 500 nM (see Methods). As most classified peptides were HLA restricted (Figure 1), and because of interest for the human immune system, we decided to restrict the analysis to these pMHCs. The positive association with immunogenicity of the large and aromatic Phenylalanine (permutation test: p<0.01), and the negative association of the small Serine (permutation test: p<0.001) were most prominent (Figure 2). In addition, significant associations with immunogenicity were observed for Isoleucine, Lysine, Methionine and Tryptophan (permutation test: p<0.05; False discovery rate (FDR) for multiple testing determined as in [46]: q<0.05). The same associations were found when pMHCs were selected based on binding affinity predictions with an alternative MHC-I binding predictor (Spearman rank test: c = 0.91; p<0.001, details are given in the Methods), or an MHC-I ligand predictor that takes into account peptide processing (Spearman rank test: c = 0.89; p<0.001, details are given in the Methods), or when pMHCs with matched predicted MHC binding affinities were selected (Spearman rank test: c = 0.95; p<0.001, details are given in the Methods).

To test if the observed associations might be the result of an underlying preference for certain amino acid characteristics, the enrichment of every amino acid in immunogenic vs non-immunogenic peptides was determined, and the enrichments were compared to physicochemical and biochemical properties described in the AAindex database [47] (see Methods). None of the amino acid properties described in AAindex (n = 505) were similar to our enrichments (Supplemental Table S2). Thus, T-cell preferences do not seem to follow a known amino acid property, possibly a combination of properties are prefered that contribute to a better interaction with the T-cell receptors. To try to unravel this combination, an analysis of amino acids grouped according to broad characteristics such as size, charge and aromaticity was performed (see Methods). For groups of amino acids with opposite characters, e.g. small and large amino acids, the number of residues in immunogenic versus non-immunogenic peptides were compared. This analysis showed that large and aromatic residues were overrepresented in immunogenic peptides presented on HLA (Fisher's test: p<0.02; see Table 1). In addition a trend for the overrepresentation of acidic residues was observed in immunogenic peptides (p = 0.06). Unfortunately, it is difficult to unravel if size and/or aromaticity was most important for immunogenicity, because amino acids share combinations of such characteristics.

**Table 1:** Amino acid characteristics of immunogenic peptides presented on HLA class I molecules.

	Total AA count		Enrichment in recognized peptides	p-value (Fisher's exact test)
	immunogenic	non-immunogenic		
large AA's	384	132	1.28	0.014
small AA's	653	304	0.94	
aromatic AA's	326	111	1.29	0.012
non-aromatic AA's	1522	699	0.95	
acidic AA's	185	67	1.21	0.06
basic AA's	147	78	0.83	
charged AA's	332	145	1.00	1.00
non-charged AA's	1516	665	1.00	

Sets of amino acids were counted in immunogenic and non-immunogenic peptides based on size, aromaticity, acidity and charge, and enrichments were determined (see Methods). The association of these characteristics (e.g. size) with immunogenicity was tested by comparing the distributions in one extreme of a characteristic with the distribution in the other extreme of that characteristic (e.g. large versus small) using Fisher's exact test. This way, one test is performed per characteristic.

Our results might be biased by the large set of HLA-A*0201 presented peptides. Therefore, we excluded all HLA-A*0201 presented peptides and repeated our analysis. This did not affect the amino acid profile in immunogenic and non-immunogenic peptides much, for every amino acid that was significantly associated with immunogenicity based on all pMHCs (F,I,K,M,S and W in Figure 2, indicated by stars), the same trend (i.e. over- or underrepresentation) was observed for the non-HLA-A*0201 presented peptides (Supplemental Figure S1). In addition, an overrepresentation of large and aromatic residues was observed in the immunogenic pMHCs. Moreover, the same results were obtained in an analysis based on only HLA-A*0201 presented peptides (Supplemental Figure S1). Thus, the observed T-cell preferences for certain amino acids were robust to either excluding or selecting the HLA-A*0201 presented peptides.

### T-cell recognition of peptide positions

The data set of immunogenic and non-immunogenic pMHCs enabled us to investigate another aspect of immunogenicity: the importance of different positions in the presented peptide. Structural studies, as well as immunogenicity studies of specific T-cell clones with altered peptide ligands, suggest that some positions in a presented peptide, especially positions 4–6, are in close contact with the TCR [40], [48], [49] and important for specific T-cell responses [38], [50]–[54]. If a certain position has a large effect on T-cell recognition, the amino acid profile at that position is expected to be different for immunogenic (i.e. T-cell recognized) compared to non-immunogenic (i.e. T-cell unrecognized) pMHCs. This difference was determined per position, using only non-anchor positions to avoid any effect of HLA binding (see Methods), i.e. by excluding positions P1, P2 and P9 for most HLA molecules, but e.g. P2, P5 and P9 for HLA-B*0801. The difference between the amino acid profiles of immunogenic and non-immunogenic pMHCs was measured using Kullback-Leibler's measure of divergence. This measure allows to estimate how well one profile can be described using the other profile, the divergence is larger if the profiles are more different from each other. The largest difference between immunogenic and non-immunogenic pMHCs was observed at positions 4, 5 and 6 (Fisher's test: p<0.01; Table 2), and a smaller, less significant difference was observed at position 7 (Fisher's test: p = 0.06; Table 2). These results are in line with previous studies on TCR-pMHC-interactions, and confirms that our data sets of immunogenic and non-immunogenic peptides carry known signatures of T-cell recognition.

**Table 2:** Position dependent differences between immunogenic and non-immunogenic peptides.

Position	Kullback-Leibler divergence
1	NA†
2	NA (anchor)
3	0.10
4	0.31 **
5	0.30 **
6	0.29 **
7	0.26 *
8	0.18
9	NA (anchor)

For peptides presented on HLA class I molecules in HLA transgenic mice that were either known to be immunogenic or non-immunogenic (see Methods), amino acids were counted per position. The 20 counts for immunogenic and non-immunogenic pMHCs were compared per position using the Kullback-Leibler divergence. A Fisher's test (Methods) was done to determine if the distributions were significantly different (* p<0.05; **p<0.01).

†Position 1 is not an anchor in every HLA molecule, nonetheless it is an anchor in most pMHCs wherefore the divergence cannot be estimated at this position.

### Predicting immunogenicity

**Figure 3:**
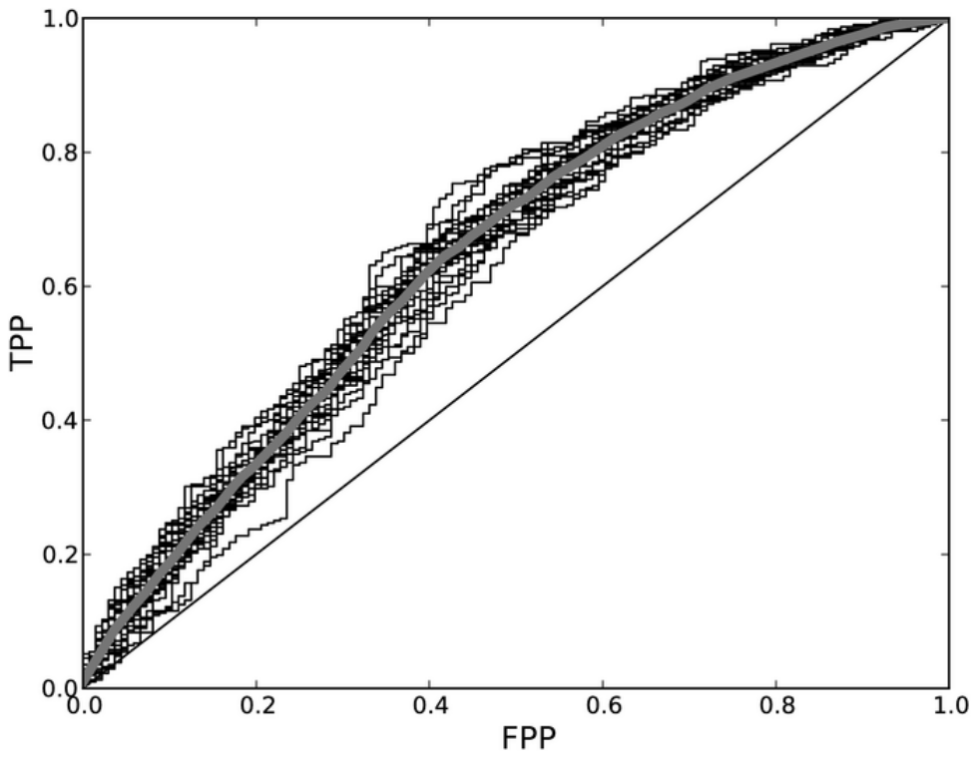
Cross-validation of the immunogenicity model. Two-thirds of the data were used for making the immunogenicity model (see methods) and one-third for cross-validation. The average ROC (thick grey line) of 25 of such cross-validations (thin lines) are plotted. The average AUC was 0.65.

Next, we tested whether the observed associations of certain amino acids with immunogenicity and the importance of different positions, were valid in other data sets. Therefore, the results presented so far were combined into a model to predict the immunogenicity of new pMHCs. In this model, the enrichment of an amino acid in immunogenic peptides, weighted by the importance of the position at which it was found, was used to score HLA class I presented peptides (see methods and Supporting Table S1). In a 3-fold cross-validation experiment, i.e. where two-thirds of the data were used for building the model and one-third for testing, could this model distinguish immunogenic from non-immunogenic peptides on HLA class I molecules with a significant accuracy: on average 66% of the immunogenic pMHCs got a positive score, compared to 44% of the non-immunogenic pMHCs (Wilcoxon rank-sum test: p<0.001; AUC = 0.65; Figure 3). Comparable prediction performances were obtained in a 10-fold cross-validation using immunogenic and non-immunogenic pMHC sets that were selected to had matched MHC binding affinities (Wilcoxon rank-sum test: p<0.05; AUC = 0.61, details are given in the Methods) or MHC binding plus processing affinities (Wilcoxon rank-sum test: p<0.05; AUC = 0.63, details are given in the Methods). Thus, the amino acid enrichments and position importances were general enough to predict, to some degree, the immunogenicity of a pMHC.

**Figure 4:**
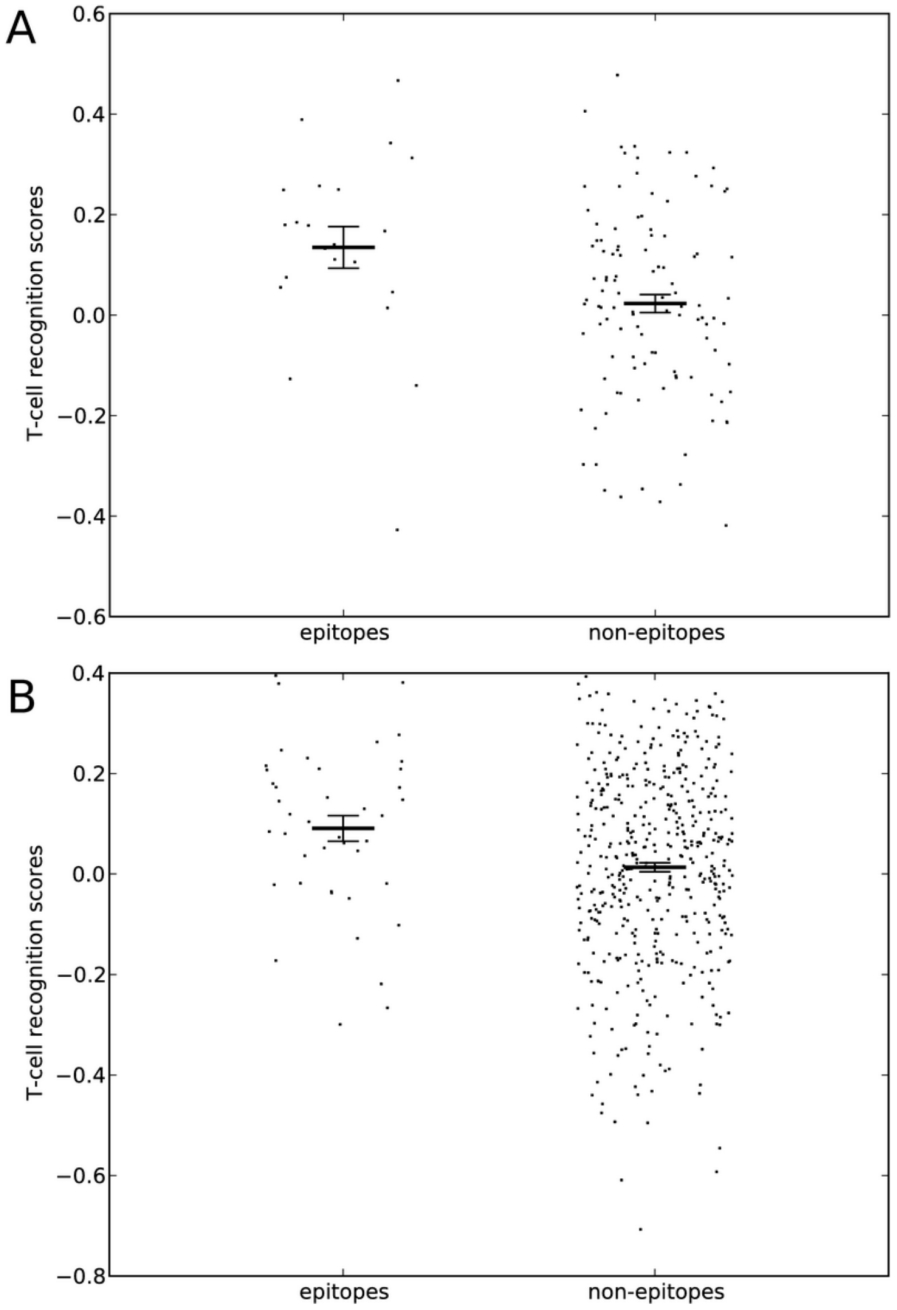
Predicting Dengue-derived CTL epitopes with the immunogenicity model. Immunogenicity scores were determined for non-redundant epitopes (n = 22) and non-epitopes (n = 110) identified in mice by Weiskopf et al. [55] (A), and for non-redundant epitopes (n = 42) and non-epitopes (n = 477) identified by Weiskopf et al. in humans [56] (B). Average and variation of the average are shown as thick lines with error bars, individual scores are shown as dots. Both in mice and in men, the epitopes had a significantly higher immunogenicity score than the non-epitopes (Murine data (A): p<0.01 (Wilcoxon rank-sum test); AUC = 0.69. Human data (B): p = 0.014 (Wilcoxon rank-sum test); AUC = 0.61).

Recently, Weiskopf et al. analyzed the immune targeting of a large number of Dengue-derived peptides presented on various HLA molecules, upon infection of HLA-transgenic mice with Dengue virus [55]. 22 non-redundant 9mer epitopes and 110 non-redundant 9mer non-epitopes with a high predicted binding affinity (<500 nM) were reported in this study [55]. This novel data set presented an opportunity to test if our observations could be extended to an independent data set. While epitopes are expected to be immunogenic, some non-epitopes may well be immunogenic in immunization experiments, but lack immune targeting in the experiments from Weiskopf et al. due to other factors such as a lack of processing or expression of the peptide during infection. Despite this problem and surpassing our expectations, the immunogenicity model scored the epitopes much higher than the non-epitopes (Wilcoxon rank-sum test: p<0.01; AUC = 0.69; see Figure 4A). Thus, this analysis further supports that certain amino acids associate with immunogenicity. In addition, this analysis demonstrates how one could apply immunogenicity predictions to enrich for epitopes in epitope discovery projects by exluding non-immunogenic pMHCs. If 38% of the Dengue-derived peptides (epitopes plus non-epitopes) would not be tested because the immunogenicity model gave them a negative score, still 86% of the epitopes would be identified, as these have a positive score. In a large study where many peptides have to be tested this means a significant fraction of the work and/or resources can be saved when using the immunogenicity model to enrich for pMHCs that are better recognized by T-cells.

### Immunogenicity: Extrapolation from mice to humans

As mentioned before, few peptide immunization studies are performed in humans, and only a single pMHC could be classified as non-immunogenic in humans, disallowing a direct comparison of amino acid enrichments and position importance scores. However, human immunogenic pMHCs could be identified (Figure 1), and the amino acid profile of these pMHCs was compared to the amino acid profile of murine immunogenic and non-immunogenic pMHCs (Figure 1). The human immunogenic pMHCs were more similar to the immunogenic pMHCs in HLA-transgenic mice (Kullback-Leibler divergence = 0.024), than to the non-immunogenic pMHCs in HLA-transgenic mice (Kullback-Leibler divergence = 0.069). Thus, it seems that immunogenic pMHCs have a similar amino acid profile in mice and men.

To further test if the immunogenicity properties that were identified in the mouse system could be extended to humans, we made use of a large epitope discovery study that was recently conducted in Dengue seropositive donors by Weiskopf et al. [56]. In this study, T-cell responses were measured in Dengue seropositive donors, to predicted MHC ligands on the HLA molecules of those donors. In total, 42 non-redundant 9mer epitopes and 477 non-redundant 9mer non-epitopes were derived from this study (see Methods for selection and redundancy reduction criteria). Similar to our result based on murine data (Figure 4A), the human epitopes had a much higher score in the immunogenicity model than the non-epitopes (Wilcoxon rank-sum test: p = 0.014; see Figure 4B). This finding confirms that studies in HLA-transgenic mice provide usefull data to understand T-cell recognition in humans, in agreement with other studies that compared the immune responses in HLA-transgenic mice and men [57].

### Immunogenicity of viral and self pMHCs

**Figure 5:**
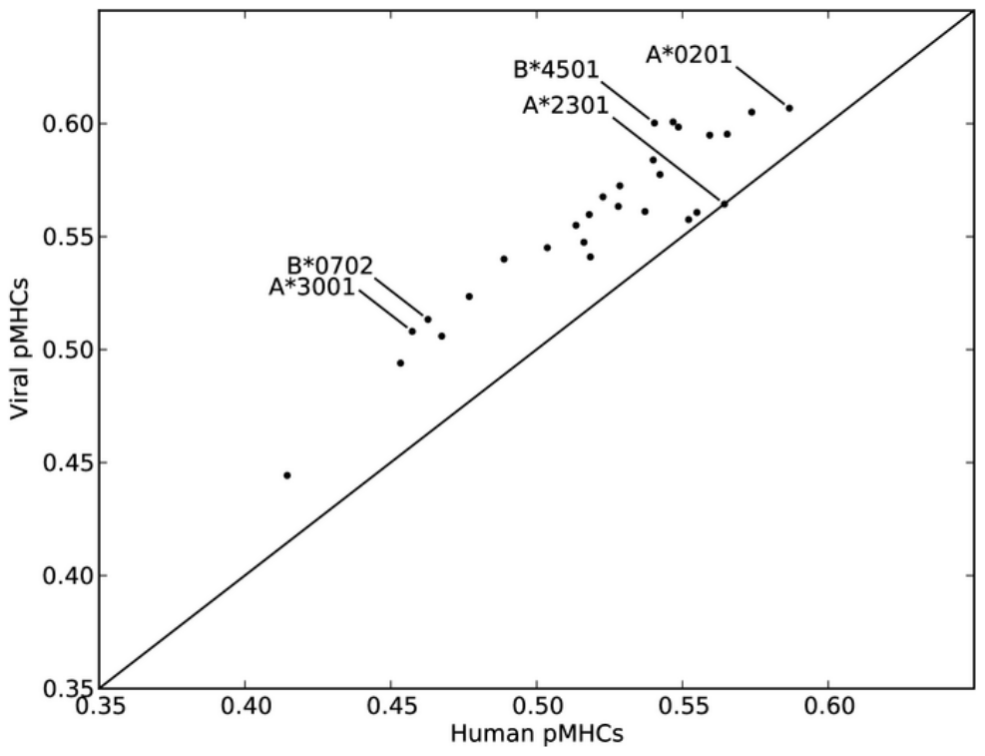
Viral pMHCs are better recognized by T-cells. For common HLA molecules (13 HLA-A and 15 HLA-B), viral and human ligands were predicted using MHC binding and precursor protein processing predictors (as in [40]). The fraction of viral pMHCs (y-axis) and human pMHCs (x-axis) with a positive score in our immunogenicity model is shown. The diagonal denotes the line y = x, HLA molecules with a larger fraction of positively scoring viral pMHCs fall above this line, which was the case for 27 of the 28 HLA molecules (sign-test: p<0.001). Three HLA molecules where the difference between the (predicted) viral and human ligands was largest (B*0702, A*3001, B*4501), and one HLA molecule where the difference between viral and human ligands was smallest (A*2301), and HLA-A*0201 are indicated in the figure.

The observed T-cell preferences could be the result of neutral evolution, where random mutations have led to certain V-D-J-segments that encode for T-cell receptors with a certain preference. Alternatively, T-cells with a TCR that better recognize pathogen-derived peptides might have been selected in the thymus, by negative selection of T-cells that strongly prefer self pMHCs, or V-D-J-segments might have been selected through evolution that encode for TCRs with a preference for pathogen-derived peptides, similar to what is observed for HLA-A molecules [58]. The immunogenicity model (Supplemental Table 1) enabled us to investigate these scenarios. For 13 HLA-A and 15 HLA-B molecules, binding ligands were predicted using MHC binding and precursor protein processing predictors, for a large set of viruses and the human proteome (data selection and ligand predictions were previously described in [40]). Next, for each HLA molecule, the predicted viral and human ligands were compared. The fraction of positive scores was higher for viral ligands than for human ligands in 27 of the 28 HLA molecules (sign-test: p<0.001, see Figure 5). The enriched immunogenicity of viral ligands was largest for HLA-A*3001, HLA-B*0702 and HLA-B*4501, where the fraction of positive scores was 11% higher for viral versus human ligands. Only for HLA-A*2301 was the fraction of positive scores slightly higher in human ligands. Thus, regardless of the presenting HLA molecule, viral ligands were predicted to be more immunogenic than human ligands, suggesting that T-cell preferences have been selected, either during thymic selection or through evolution, to favour the recognition of foreign peptides.

## Discussion

Immunogenicity (i.e. T-cell recognition) is an important factor that determines if a pMHC can be targeted in an immune response. We showed that pMHCs are more likely to be immunogenic if they contain certain amino acid residues. More precisely, the presence of large and aromatic residues seemed to be associated with immunogenicity. In addition, positions 4–6 of the presented peptide were shown to have a large effect on immunogenicity. We combined these findings into a simple model and demonstrated that these observations can be extended to other data sets in both humans and mice, and used to predict the immunogenicity of new pMHCs.

Previously, other groups have studied the importance of different positions in an MHC-I presented peptide using two distinct approaches. First, specific T-cell clones have been assessed for the recognition of variant peptides [38], [50]–[54], [59], and most T-cell clones in such studies lost the recognition of peptides that were substituted at positions between the anchors (P3–8). In well-studied systems, such as the T4 T-cell clone recognizing the SLFNTVATL peptide on HLA-A2, recognition of position P5 was most specific, followed by a high specificity at the flanking positions P4 and P6 [38], [51]. Second, the study of TCR-pMHC structures contributed to the understanding of immunogenicity. In such structures, the number of interactions between the TCR and different positions of the MHC-I presented peptide have been evaluated [40], [48], [49], and more interactions were observed with the positions P4–P8. The results from both approaches seem to agree: that positions P4–8, and of those especially positions P4–6, are most important for immunogenicity. Here, we find that the amino acids at different positions of the MHC-I presented peptide in immunogenic and non-immunogenic pMHCs differ most at positions P4–P6, and less so but significantly at P7. Thus, our findings are in agreement with the previous observations, and present a third line of evidence that positions P4–P6 are most important in the TCR-pMHC-interaction.

The effect of P1, P2 and P9 on T-cell recognition could not be analyzed as these positions determine peptide binding in most HLA molecules. Similarly, TAP transport and proteasome cleavage might bias our conclusions on the importance of different positions for immunogenicity. TAP has been shown to have specificity at the C-terminus of a peptide (P9) and at the three N-terminal positions (P1, P2 and P3 of the MHC-I presented peptide if aminopeptidase activity is ignored) [24]. For proteasome cleavage activity, specificity is strongest at positions next to the cleavage site, i.e. corresponding to position P9 and to a much smaller extend P8, of the MHC-I presented peptide [25]. Thus, neither TAP transport nor proteasome cleavage preferences are expected to significantly affect positions P3–8, therefore we think that an effect of these processes on our position importance analysis can be ruled out. If any, an effect might be present at P3 (for TAP) or P8 (for the proteasome), but the measured importance of these positions was smallest (Table 2).

We focused in this paper on MHC-I presented peptides and showed a preference for certain amino acids, especially those with large, aromatic residues. This fits with a previous study by Alexander et al. who tested the immunogenicity of so-called PADRE peptides, that are presented on most MHC class II molecules but that differ in T-cell recognition sites [60]. Interestingly, they showed that PADRE peptides with large residues are very immunogenic. Thus, T-cell preferences for peptides presented either on MHC class I or II molecules seem to be similar. We have also performed an analysis of amino acid preferences for H-2 restricted pMHC complexes on a limited dataset, and found a different pattern of enrichment scores for the amino acids that does not correlate with the enrichment scores we obtained while using HLA restricted pMHC complexes (Spearman rank test: c = −0.06; p = 0.80). However, an association with immunogenicity of both aromatic and large amino acids was also found for H-2 restricted pMHC complexes (Fisher's exact test: p<0.05, both). The difference might be expected given the altered peptide binding preferences of H-2 molecules, that present short 8mer peptides and use more and different auxiliary anchor positions than HLA class I molecules [61]. Currently, the limited number of non-immunogenic H-2 restricted pMHCs (n = 46, Figure 1) prohibits us to draw conclusions on the difference between H-2 and HLA restricted pMHCs. Therefore, more experimental data and further studies are necessary to analyze if the differences are significant, and if so, if they are due to structural, evolutionary, or other differences between the immunogenicity of peptides that are presented on HLA class I or H-2 molecules. Similarly, the immunogenicity of peptides might be different when they are presented on different HLA molecules, though to a lesser degree as different HLA molecules are more comparable to each other than to H-2 [61]. When more data would be available, the influence of MHC-I restriction on immunogenicity could be investigated.

Based on the comparison of immunogenic and non-immunogenic pMHCs we derived a simple model to predict immunogenicity. We call our immunogenicity model simple because it does not account for non-linear influences on immunogenicity, or position-specific amino acid enrichment scores. Position-specific scores (i.e. 20 enrichment scores per position) seem to present an opportunity for further improvement, as different preferences seem to occur at different positions, e.g. a preference at position 6 for non-charged residues (Fisher's exact test: p<0.05; not shown). However, the current data sets are too small to investigate the preferences at each position separately, or to incorporate position specific preferences into the immunogenicity model without running the risk of overfitting. Especially data from non-immunogenic pMHCs is lacking as a result of the preferential reporting of positive results. We believe our simple model provides a proof-of-principle that immunogenicity is predictable, and that more complex and possibly more accurate predictors can be made if more data, especially non-immunogenic pMHCs, is available.

We benchmarked our immunogenicity prediction model on epitope and non-epitope data sets that were derived from mice and men. As expected, most epitopes obtained high scores in our model. Conversely, some non-epitopes do not elicit an immune response because they are non-immunogenic, indeed some of the non-epitopes scored very low in our immunogenicity prediction model. However, non-immunogenicity is only one of several reasons that can explain why a peptide is not immune targeted (i.e. is a non-epitope). For instance, a lack of expression or processing of the precursor protein, or regulation by Tregs might cause certain peptides to be non-epitopes. An understanding of all these processes and how to combine them will be necessary for improved epitope/non-epitope predictions. Nevertheless, the prediction of non-immunogenic peptides will be usefull in future large-scale epitope discovery studies, as it shortens the list of potential peptides that have to be tested without finding less epitopes. In both data sets that were used to test the immunogenicity prediction model, we showed that ∼40% of the candidate peptides can be discarded, while losing only 15–30% of the epitopes. Even though this might seem like a small improvement, the effect can be large in studies where patient-derived samples or other resources are limited.

The group of Ho et al. have pioneered the field of immunogenicity predictors, and recently published a method for immunogenicity prediction called POPISK [62]. POPISK aims to predict the immunogenicity of HLA-A*0201 presented peptides and reports a high accuracy in cross-validation (AUC = 0.74, see [62]). POPISK is different from our predictor in three important ways. First, it is trained on all peptide positions of HLA-A*0201 presented peptides, whereas we exclude positions that influence the binding affinity such as the anchor positions P2 and P9. Second, non-immunogenic pMHCs in the IMMA2 data set that was used to train and test POPISK were not defined based on negative results in a peptide-immunization experiment, therefore other explanations for the absence of an immune response besides non-immunogenicity cannot be excluded. Third, POPISK is a rather complex model using support vector machines and string kernels. A complex model runs the risk to be overtrained, especially on a limited data set, which will not be noticed in cross-validation if redundant peptides are not excluded from the data sets, as is the case for the IMMA2 data set that was used to build POPISK [62]. Possibly due to such differences, POPISK is not able to score the Dengue-derived epitopes that were recently published by Weiskopf et al. [55] higher than the non-epitopes, neither based on all pMHCs (1-sided t-test: p = 0.28; Supplemental Figure S2), nor on the HLA-A*0201 presented pMHCs (1-sided t-test: p = 0.39; Supplemental Figure S2). A model like POPISK might perform better if it is trained on more high quality data. For now, we think that the available data only permits the construction of simple proof-of-principle immunogenicity predictors, and the study of basic features of immunogenicity.

The TCR repertoire can be influenced by the hosts genetics, e.g. the HLA-background of a host and thymic selection [63]–[65], or the likelyhood of certain VDJ-recombinations [65]–[68]. Even though the T-cell pool might vary in every individual as a result of such influences, we found that T-cells have a preference for certain amino acids (see Supplemental Table S1, the immunogenicity model). That preferences are similar among hosts agrees with the observation from Alanio et al. that T-cell precursor frequencies for the same pMHC are similar in different hosts, whereas precursor frequencies for different pMHCs vary substantially [6]. Furthermore, we showed that these preferences resulted in a better recognition of pathogen-derived pMHCs (Figure 5). The observed preferences might be the result of natural selection for the increased immunogenicity of pathogen-derived pMHCs, additional to the widely suggested selection for TCR-genes that interact with conserved MHC-I motifs [49], [69]–[71]. Alternatively, T-cells might be selected in the thymus to have a preference for nonself pMHCs. In this scenario, strong thymic selection would take out self-recognizing T-cells, that might share a preference for amino acids that are more abundant in the human proteins. It would be very interesting to measure if the T-cell preferences that are described here, are present before or only after thymic selection.

Thus far, we described the immunogenicity of a pMHCs as an inherent feature, caused only by pMHC specific factors such as the interactions with the TCR repertoire. However, factors outside the TCR repertoire and the specific pMHC might also play a role. For instance, when different pMHCs interact with the same part of the TCR repertoire they could compete with each other. Some peptides might face a stronger competition, e.g. if they are composed of more general amino acids. For that reason, a possibly high precursor frequency that would have been measured for a single pMHC, should not per definition translate in a high immunogenicity when this pMHC is presented in the context of an infection among other pMHCs.

The identification of all pMHCs that are epitopes would be prerequisite to a complete understanding of the cellular immune responses. That understanding would help the study of host-pathogen interactions, for instance how pathogens try to escape from immune recognition by mutating the epitopes that are under pressure of the immune system [72], [73]. In addition, the identification of epitopes will help the development of better vaccines, that effectively elicit protective immune responses. In past years, investigations of the MHC-I presentation pathway led to the development of highly accurate predictors that can predict which pMHCs are formed upon infection. However, we know very little on which presented pMHCs are used by the immune system to mount a T-cell response. Previously, we and others showed that self-similarity plays an important role in excluding some pMHCs as potential epitopes [37], [38], [40], and we estimated that at least one-third of the foreign pMHCs would be ignored to prevent otherwise autoimmune responses [40]. Now, we add another piece to the epitope-puzzle, and show that immunogenicity is to some degree predictable. A combination model that integrates predictions from the MHC-I presentation pathway, self-overlaps and immunogenicity might help to more accurately predict epitopes in the future, and to assist large-scale epitope discovery projects.

## Methods

### Ethics statement

Ethics approval was granted for the dengue virus large scale epitope discovery study from the LIAI IRB and the Ethical Review Committee at Medical Faculty, University of Colombo, Sri Lanka.

### Generation of data sets

The aim of this study is to compare immunogenic and non-immunogenic peptides on MHC class I molecules. These peptides were obtained from data sets from Assarsson et al [43], Kotturi et al. [44] and an unpublished data set on Coxiella Burnetti-derived peptides as well as from the IEDB [45]. Only 8–10mer peptides were selected, for which reliable MHC-I binding predictions are possible. Peptide MHC-I binding affinities were predicted using NetMHC-3.2 [10], the best performing predictor according to a large benchmark study [8]. Only pMHCs with a high predicted binding affinity were included (<500 nM). Two other MHC-I binding predictors were used, NetMHCpan-2.4 [13], [14] and NetCTL-1.2a [10], to make MHC-I binding predictions, classify pMHCs, and to redo the enrichment analysis. In each case, near-identical enrichment scores were observed. In the analysis with NetCTL-1.2a, MHC-I ligands were predicted based on a combination of prediction scores for proteasome cleavage, TAP transport and MHC-I binding. Standard settings of NetCTL-1.2a were used to combine the scores and to discriminate ligands from non-ligands [10].

The data set by Assarsson et al of vaccinia-derived peptides presented in an HLA-A*02 transgenic mouse model, has been classified by the authors into “dominant”, “subdominant”, “cryptic” and “negatives” [43]. We classified peptides as immunogenic if they induced a positive response in the peptide-immunization experiments (categories “dominant”, “subdominant” or “cryptic”; n = 63; see Figure 1), while a peptide with a negative response was classified as non-immunogenic (category “negative”; n = 33; see Figure 1).

Data described by Kotturi et al. was kindly provided by the authors. Kotturi et al. studied the immunogenicity of peptides presented on HLA-A*1101 that are derived from Arenaviruses [44]. In HLA-transgenic mice, T-cell recognition upon peptide-immunization was measured. If a significantly high T-cell response was elicited (t-test: p<0.05; SFC>20 per million; stimulation index>2.0) in at least two independent measurements (detailed in [44]), we classified a peptide as immunogenic (n = 116, see Figure 1). All other peptides were classified as non-immunogenic (n = 159, see Figure 1).

A previously unpublished set of peptides derived from Coxiella burnetti proteins was tested for immunogenicity in wild type Bl/6 mice. The immunization protocol and criteria for positivity were the same as for the Kotturi data set [44]. 11 Immunogenic and 16 non-immunogenic pMHCs were derived from this experiment.

A large data set was derived from the IEDB, where all T-cell response experiments (i.e. peptide-immunizations, vaccination and infection experiments) with MHC class I presented peptides in mice and humans were downloaded (www.iedb.org [45]). All entries from HLA-A*1101-transgenic mice were excluded, to rule out any bias resulting from the incompatibility of the HLA-A*1101 binding motif and the preferences of murine TAP [74]. This requirement was alleviated for the data from the Kotturi study as we know that in this peptide-immunization study there was no need for peptides to be TAP transported. If the restricting MHC-I molecule was not reported, it was estimated from the reported mouse strain MHC-I background; if multiple MHC class I molecules were possible the molecule with highest predicted binding affinity was selected as the restricting MHC-I molecule. Immunogenic pMHCs were selected based on a reported positive T-cell response, and the absence of restimulation *in vitro*. Non-immunogenic peptides were selected based on a reported negative T-cell response and the absence of any reported positive T-cell response. In addition, as with the other data sets, non-immunogenic pMHCs were required to be identified in a peptide-immunization experiment. Therefore, the following criteria were applied: the antigen-epitope relation had to be “epitope”, meaning that only the epitope was used for stimulation and not for instance the complete pathogen, and the first in vivo immunogen had to be “peptide from protein”, meaning a peptide immunization study was performed. This resulted in the identification of 2029 immunogenic and 152 non-immunogenic pMHCs (see Figure 1). As only peptides of the same length were studied here, 9mers were selected, for which most pMHCs were available. All selected pMHCs are listed in Dataset S1.

### Generation of non-redundant data sets

The data in databases such as the IEDB is biased towards pMHCs that are well-studied. For instance, for the SIINFEKL peptide we find 358 entries in the IEDB, and 22 entries of single amino acid mutants. To eliminate such cases in our dataset, a redundancy reduction based on source protein mapping was applied. First, for all peptides in our datasets that were identified as immunogenic or non-immunogenic following the above requirements (see Figure 1), source proteins were downloaded via the sequence information provided in the IEDB. In addition, for the Vaccinia-, Coxiella- and Arenavirus-derived pMHCs, the proteomes of these viruses were downloaded via EBI/EMBL in July 2011. Next, all peptides were mapped to all source proteins using BLASTP 2.2.18 [75], and a mapping was considdered successful if more than 75% of the residues matched. Two peptides were defined as redundant if more than half of their residues map to the same positions in any of the source proteins. In addition, all peptides that could not be mapped to a source protein were discarded. Redundant peptides were filtered out, wherein we prioritized the selection of pMHCs with more entries in the IEDB. If redundant pMHCs with equal priority remained, the selection of one of them was based on chance; this was the case for only 5.7% of the non-immunogenic pMHCs and 4.4% of the immunogenic pMHCs. This procedure generates pMHC sets that can vary slightly. A single non-redundant pMHC set was selected and used for the presented analysis, but every result was tested and repeated in ten (of ten) non-redundancy selections.

### Selecting Dengue-derived epitopes in mice and men

In mice, Weiskopf et al. analyzed the immune targeting of a large number of Dengue-derived peptides presented on HLA-A*0101, HLA-A*0201, HLA-A*1101 and HLA-B*0702, upon infection of HLA-transgenic mice with Dengue virus [55]. pMHCs with a 9mer and a high predicted binding affinity (<500 nM) were selected from this study [55]. When selecting non-redundant peptides, the selection of epitopes with a high T-cell response and non-epitopes with a strong binding affinity was prioritized. Selected epitopes (n = 22, Dataset S2) and non-epitopes (n = 110, Dataset S2) did not differ significantly in their predicted binding affinities.

In humans, Weiskopf et al. tested the immune responses to Dengue-derived peptides in Dengue seropositive donors [56]. For every donor, the HLA background was determined, and peptides predicted to be presented on these HLA molecules were tested. We defined pMHCs with a positive immune response in any of the donors as epitopes; a pMHC that never evoked an immune response and that was not redundant with an epitope was defined as a non-epitope. Only non-redundant 9mer peptides from the epitope and non-epitope sets were selected. In addition, as 5 of the 229 donors contributed to 50% of all detected immune responses, we selected per donor 5 epitopes with highest immune responses, to prevent a bias that might have been caused due to the very broad T-cell response in these donors. Selected epitopes (n = 42, Dataset S2) and non-epitopes (n = 477, Dataset S2) did not differ significantly in their predicted binding affinities.

### The immunogenicity model

The immunogenicity model is build based on the enrichment of amino acids in immunogenic versus non-immunogenic peptides and the importance scores of different positions of the MHC-I presented peptide (Table 2). For each MHC-I molecule, the impact on binding affinity was determined per position of the presented peptides (as explained in [40]). The six positions with least impact on the binding affinity were defined as non-anchor positions, these six positions can differ for different MHC-I molecules that use different anchor positions. Only non-anchor positions were used to study differences in immunogenicity, as anchor positions might reflect a difference in binding affinity rather than a difference in immunogenicity. Per amino acid, the enrichment is calculated as the ratio between the fraction of that amino acid in the immunogenic versus non-immunogenic data sets. For instance, Tyr occurs with a frequency of 2.5% in immunogenic and 1.5% in non-immunogenic peptides, the enrichment in immunogenic peptides is 1.7-fold, and the natural logarithm of this enrichment is 0.54. We call this enrichment the log enrichment score. To predict the immunogenicity of a new pMHC, per non-anchor residue of the presented peptide the log enrichment score was found and weighted according to the importance of that position (measured as the Kullback-Leibler divergence; see Table 2). The weighted log enrichment scores of all (non-anchor) residues were summed, the resulting score was termed the immunogenicity score. The larger the immunogenicity score, the more the pMHC is like the immunogenic peptides and therefore expected to be immunogenic. The log enrichment scores of amino acids at anchor residues are masked, i.e. not used to derive the immunogenicity score. These assumptions resulted in the following formula to calculate the immunogenicity score, S, of a peptide ligand, L, presented on an HLA molecule, H:
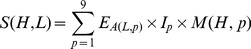
(1)Where for every position p in the ligand L, the log enrichment score E for the amino acid at that position A(L,p) weighted by the importance of that position 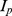 is summed. The eventual masking of anchor positions on that HLA is obtained by setting M(H,p) to 0.

The immunogenicity score model was tested in a 3-fold cross-validation experiment, where a random two-thirds of the data was used to calculate the log enrichment scores. These log enrichment scores, together with the position importance weights (Table 2) were then used to construct the immunogenicity score model as described above, and the other one-third of the data was used to test its performance. 25 Cross-validations were performed. Our final immunogenicity score model, that is used throughout this paper, is based on all non-redundant HLA class I presented peptides found in HLA-transgenic mice. As the selected non-redundant set of peptides varies slightly (explained above), the final model was constructed by repeating the non-redundancy selection and model building 100 times, and taking average log enrichment scores per amino acid from these 100 models. The final log enrichment scores, position importance weights and explanations on constructing the immunogenicity score model are given in Supplemental Table S1.

### Amino acid properties

Different groups of amino acids were assembled based on shared characteristics. These groups were used to test if certain characteristics associate with immunogenic or non-immunogenic peptides. Small amino acids were defined as having a size of less than 120 Da (A,G,P,S,T,V), large amino acids as having a size of more than 150 Da (F,H,R,W,Y). Definitions of the other groups were based on conventional views: Aromatic amino acids (F,H,W,Y), non-aromatic amino acids (all amino acids that are not aromatic), charged amino acids (D,E,H,K,R), non-charged amino acids (all amino acids that are not charged), acidic amino acids (D,E) and basic amino acids (H,K,R). For opposite characteristics, e.g. large versus small, the enrichment of amino acids with a certain characteristic, e.g. large, was determined by comparing the ratio of large amino acids in immunogenic versus non-immunogenic peptides with the ratio of all amino acids in immunogenic versus non-immunogenic peptides.

From the AAindex database [47], all (n = 505) amino acid properties were downloaded in March 2012. In this database, similar properties are defined by their strong correlation (Spearman-rank test: absolute correlation coefficient >0.8).

### Creating sets of binding affinity matched pMHCs

Two sets of pMHCs with matching predicted binding affinity scores were created by making bins of scores, and selecting the maximum number of pMHCs from each set such that the distibutions over the bins in each set was the same. The first bin encompassed all pMHC with a binding affinity lower than 1 nM. The other bins were separated by five values that were chosen on a logarithmic scale from 1 nM to 500 nM, i.e. 1 nM, 4.7 nM, 22.4 nM, 106 nM and 500 nM. Two sets of pMHCs with matched processing probabilities and matched MHC binding affinities were created in a similar way using NetCTL prediction scores (encompassing MHC binding and peptide processing propensity scores). Hereby, to evaluate all scores on a logarithmic scale, the scores were increased with 1.1625 such that the minimum score was higher than 1.0. The bins were separated by five values that were chosen on a logarithmic scale from 1 to 5. In all cases, the difference in affinity scores between the selected matched sets was tested, and shown to be not significantly different (Wilcoxon rank-sum test: p>0.05).

### Statistics

Statistical tests were performed using the stats-package from the scipy-module in Python. To assess the significance of the association of a certain amino acid with immunogenicity, a permutation test was performed. For each amino acid, first the frequency in non-anchor positions of immunogenic and non-immunogenic peptides, and the background frequency in source proteins was determined (data used for Figure 2). Next, based on the background frequency and the total number of amino acids, a random sample of immunogenic and non-immunogenic amino acids was drawn. The frequency of the amino acid in the immunogenic and non-immunogenic drawings was determined, and the difference between these frequencies was compared with the difference in the real peptides. 10000 of these permutations were performed, and the fraction of permutations in which the (permutated) difference was larger or equal than the real difference determined the probability of finding our result by chance, i.e. the p-value. Q-values, to estimate the False Discovery Rate (see [46]), were determined using the QVALUE software that is developed by Storey et al. [46]. The Fisher's test to determine if amino acid distributions were significantly different was performed in R [76]. Hereby, the Fisher's test was done with asymptotic chi-squared probabilities if the “Cochran conditions” (no cell has count zero, at least 80% of the cells have 5 or more counts) were satisfied [76], [77].

## Supporting Information

Dataset S1 **A table with all immunogenic and non-immunogenic pMHCs that were found in the IEDB, Vaccinia, Arena and Coxiella data sets (** **Methods** **).** On each row, the peptide sequence (column A), the presenting MHC molecule (column B), the host (column C) and the immunogenicity (column D) are described.
(XLS)

Dataset S2 **A table with all non-redundant murine and human Dengue epitopes and non-epitopes (** **Methods** **).** On each row, the peptide sequence (column A), the presenting MHC molecule (column B), the epitope/non-epitope classification (column C) and the host (column D) are described.
(XLS)

Figure S1 T-cell preferences for different amino acids in HLA-A*0201 presented peptides (left panel) or peptides presented on other HLA molecules (right panel). The fraction of an amino acid in immunogenic (left bar, filled) and non-immunogenic (right bar, unfilled) peptides is shown. The background frequency for each amino acid in the protein sequences that were sources of the immunogenic or non-immunogenic peptides is shown by a grey line.
(TIF)

Figure S2 **Predicting Dengue-derived CTL epitopes with the POPISK model** [**62**] **.** POPISK scores were determined for non-redundant epitopes (n = 22) and non-epitopes (n = 110) identified in mice by Weiskopf et al. [55] (left panel), and for the epitopes (n = 7) and non-epitopes (n = 31) in this set that were HLA-A*02:01 restricted (right panel). Average and variation of the average are shown as thick lines with error bars, individual scores are shown as dots. In both sets, the epitopes and non-epitopes had similar POPISK scores (All (left panel): p = 0.28 (1-sided t-test); AUC = 0.52. HLA-A*02:01 restricted (right panel): p = 0.39 (1-sided t-test); AUC = 0.49).
(TIF)

Table S1 **The immunogenicity model.** The immunogenicity score, S, is derived by summing the log enrichment scores of amino acids that are found at non-masked positions, weighted by the importance of that position (see formula and Methods). The final log enrichment scores for all amino acids are given in the left table, importance scores for the different positions are shown in the right table (also shown in table 2). An example to calculate the score for HLA-A*0201:SLFNTVATL is given.
(TIF)

Table S2 **Amino acid characteristics that correlate with our enrichment values (Supplemental Table S1).** For all amino acid indices that are described in the AAindex-database [47], the Spearman Rank correlation with enrichment scores in immunogenic pMHCs was determined. All significant (p<0.05) correlations are reported. Q-values, reported in the fifth column, give the estimated False Discovery Rate (see [46]) which was very high in all cases >0.4 due to the large number of tests performed (n = 505). The “hydrophobicity coefficient in RP-HPLC”-index showed the best correlation, but is not the only measure of hydrophobicity. All other indices with the term “hydrophobic” or “hydrophobicity” in their description (n = 35) were not significantly correlated with our enrichment scores (p>0.1, not shown).
(TIF)
