# Supplementary figures and images for: Properties of MHC Class I Presented Peptides That Enhance Immunogenicity
Source: PLoS Comput Biol. 2013 Oct 24;9(10):e1003266. doi: 10.1371/journal.pcbi.1003266 (PMC3808449; doi:10.1371/journal.pcbi.1003266)

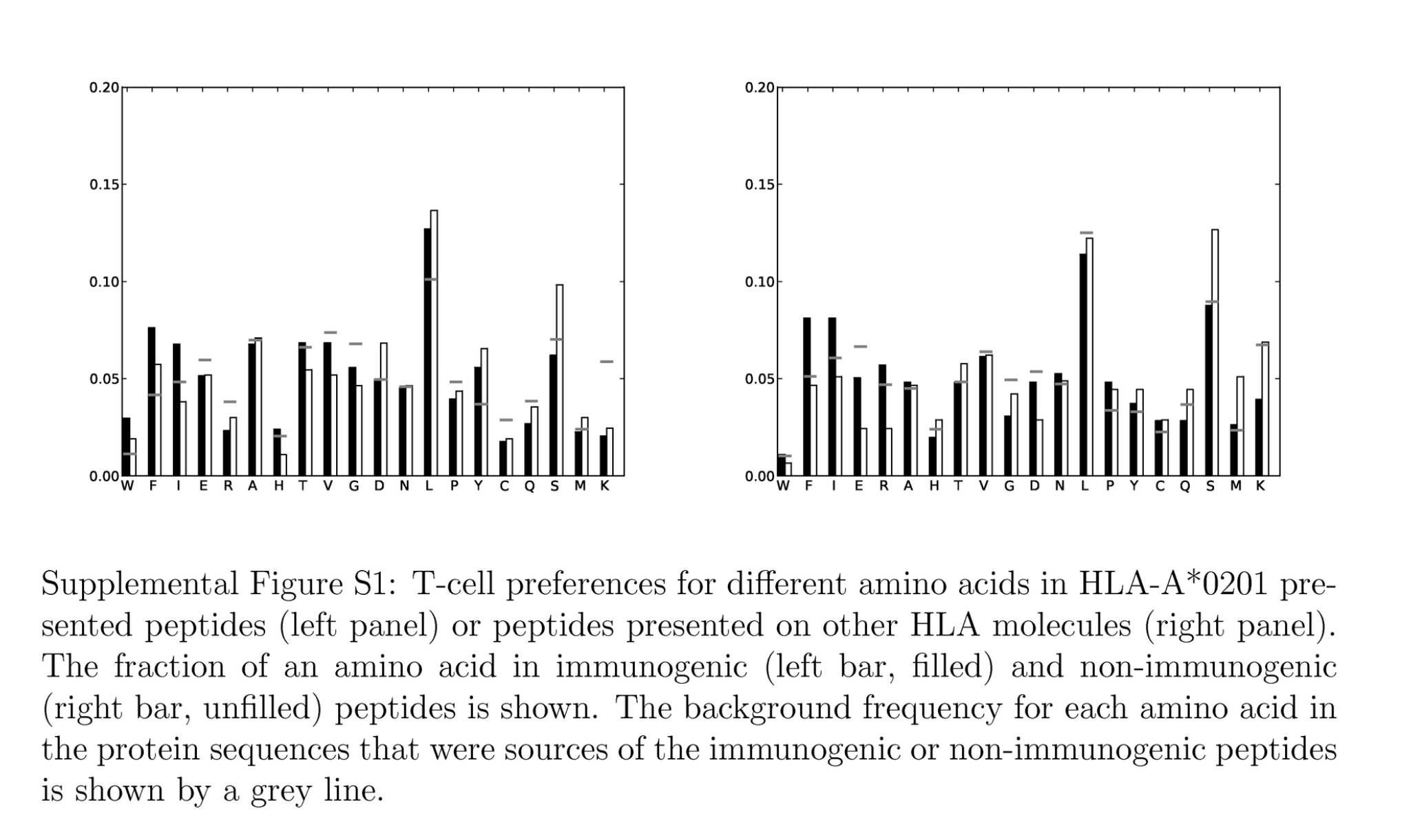

Supplement: Figure S1 — T-cell preferences for different amino acids in HLA-A*0201 presented peptides (left panel) or peptides presented on other HLA molecules (right panel). The fraction of an amino acid in immunogenic (left bar, filled) and non-immunogenic (right bar, unfilled) peptides is shown. The background frequency for each amino acid in the protein sequences that were sources of the immunogenic or non-immunogenic peptides is shown by a grey line. (TIF) [file pcbi.1003266.s003.tif]

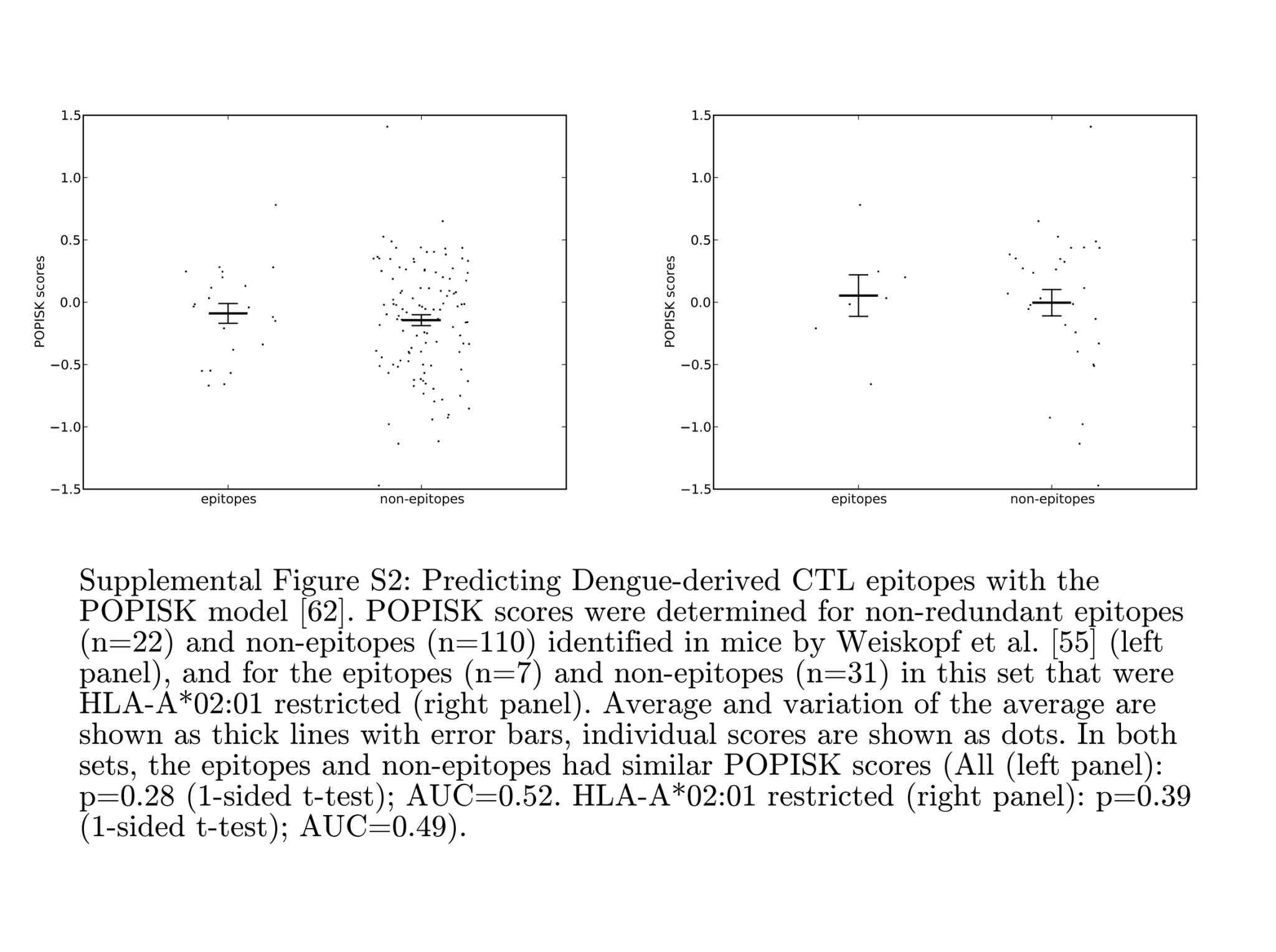

Supplement: Figure S2 — Predicting Dengue-derived CTL epitopes with the POPISK model [62] . POPISK scores were determined for non-redundant epitopes (n = 22) and non-epitopes (n = 110) identified in mice by Weiskopf et al. [55] (left panel), and for the epitopes (n = 7) and non-epitopes (n = 31) in this set that were HLA-A*02:01 restricted (right panel). Average and variation of the average are shown as thick lines with error bars, individual scores are shown as dots. In both sets, the epitopes and non-epitopes had similar POPISK scores (All (left panel): p = 0.28 (1-sided t-test); AUC = 0.52. HLA-A*02:01 restricted (right panel): p = 0.39 (1-sided t-test); AUC = 0.49). (TIF) [file pcbi.1003266.s004.tif]

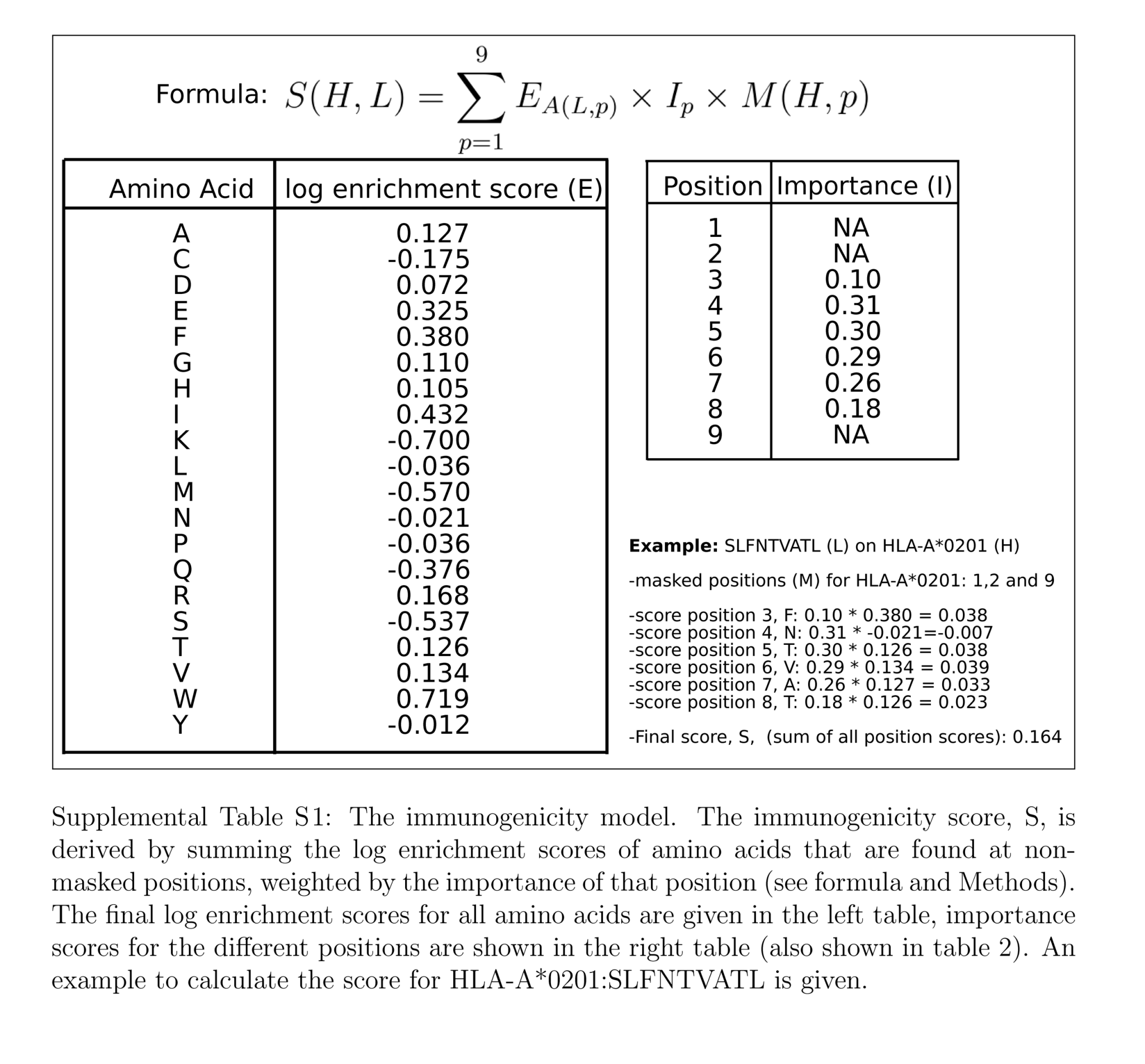

Supplement: Table S1 — The immunogenicity model. The immunogenicity score, S, is derived by summing the log enrichment scores of amino acids that are found at non-masked positions, weighted by the importance of that position (see formula and Methods). The final log enrichment scores for all amino acids are given in the left table, importance scores for the different positions are shown in the right table (also shown in table 2). An example to calculate the score for HLA-A*0201:SLFNTVATL is given. (TIF) [file pcbi.1003266.s005.tif]

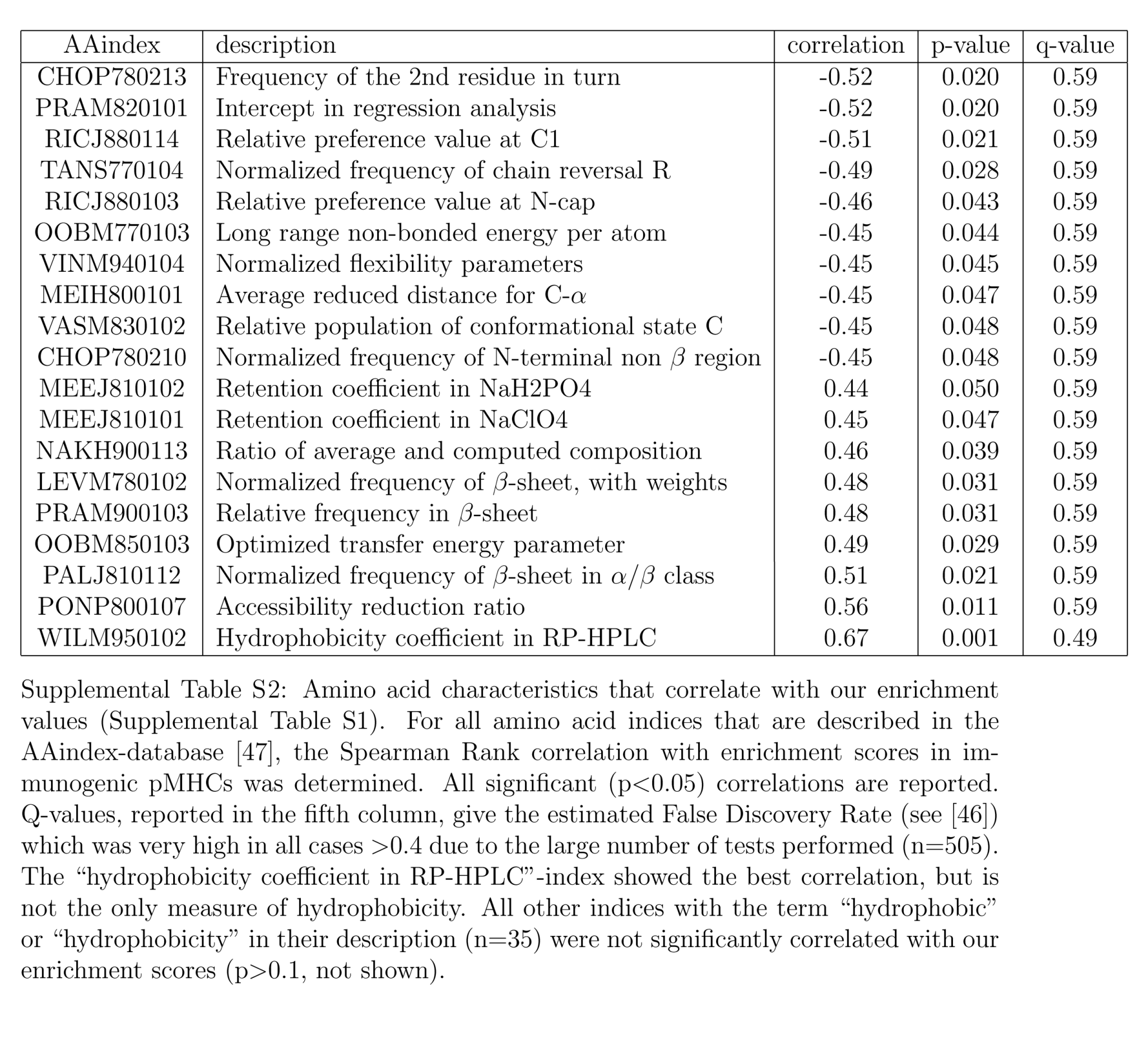

Supplement: Table S2 — Amino acid characteristics that correlate with our enrichment values (Supplemental Table S1). For all amino acid indices that are described in the AAindex-database [47], the Spearman Rank correlation with enrichment scores in immunogenic pMHCs was determined. All significant (p<0.05) correlations are reported. Q-values, reported in the fifth column, give the estimated False Discovery Rate (see [46]) which was very high in all cases >0.4 due to the large number of tests performed (n = 505). The “hydrophobicity coefficient in RP-HPLC”-index showed the best correlation, but is not the only measure of hydrophobicity. All other indices with the term “hydrophobic” or “hydrophobicity” in their description (n = 35) were not significantly correlated with our enrichment scores (p>0.1, not shown). (TIF) [file pcbi.1003266.s006.tif]
